# Assessment of Body Composition and Dietary Intake in Nursing-Home Residents: Could Lessons Learned from the COVID-19 Pandemic Be Used to Prevent Future Casualties in Older Individuals?

**DOI:** 10.3390/nu13051510

**Published:** 2021-04-29

**Authors:** Irena Keser, Selma Cvijetić, Ana Ilić, Irena Colić Barić, Dario Boschiero, Jasminka Z. Ilich

**Affiliations:** 1 Laboratory for Nutrition Science, Faculty of Food Technology and Biotechnology, University of Zagreb, Pierottijeva 6, 10000 Zagreb, Croatia; ikeser@pbf.hr (I.K.); ailic@pbf.hr (A.I.); icolic@pbf.hr (I.C.B.); 2 Department of Occupational and Environmental Medicine, Institute for Medical Research and Occupational Health, Ksaverska Cesta 2, 10000 Zagreb, Croatia; cvijetic@imi.hr; 3 BioTekna., 30020 Venice-Marcon, Italy; dario.boschiero@biotekna.com; 4 Institute for Successful Longevity, Florida State University, 1107 West Call Street, Tallahassee, FL 32306, USA

**Keywords:** bone, muscle mass, body fat, older adults, osteopenic/osteoporotic adiposity, sarcopenic adiposity, osteosarcopenic adiposity, T-score, S-score

## Abstract

The population of older adults, especially those living in the nursing homes, is growing. The sedentary lifestyle and possible poor nutrition in nursing homes place residents (NHRs) at risk for body composition impairments, malnutrition, and, subsequently, numerous chronic diseases. The aim of this study was to assess body composition (including body fluids) and dietary intake in NHRs. The association between osteosarcopenic adiposity syndrome (OSA) and its components, osteopenic adiposity (OA), sarcopenic adiposity (SA), and adiposity-only (AD), and specific macro- and micro-nutrients was evaluated as well. The study included 84 participants (82.1% women), aged 65.3–95.2 years. Body composition was assessed with an advanced bioelectrical impedance device BIA-ACC^®^ and dietary intake was assessed via 24-h recall and analyzed using “Nutrition” software. The majority (95%) of participants were overweight with a high body fat and low muscle and bone mass, leading to a high prevalence of OSA (>50%), OA (13%), and AD (26%). There were only a few participants with SA, and they were not analyzed. The highest extracellular water/total body water ratio was observed in the OSA participants, indicating a heightened inflammatory state. Participants in all three body composition categories had a similar nutrient intake, with protein, fiber, omega-3 fatty acids, and almost all micronutrients being far below recommendations. In conclusion, a high prevalence of OSA among NHRs accompanied by a poor dietary intake, could place these residents at a very high risk for COVID-19 infections. Therefore, optimization of body composition and nutritional status should be included along with standard medical care in order to provide better health maintenance, particularly in the COVID-19 era.

## 1. Introduction

Older adults are the fastest growing segment of the population throughout the world [1]. In the European Union (EU), the number of individuals aged 65 years and older is projected to increase from 17.5% in 2011 to nearly 30% by 2060, while the population of those aged 80 years and older is predicted to triple during this period [2]. Similar statistics have been reported in many individual EU countries, including Croatia [3]. This rise in the number of older adults leads to a subsequent increase in numerous chronic conditions, including body composition disorders, which are recognized as a major driver to many serious diseases (diabetes, cancers, and obesity) and are also linked to increased susceptibility to COVID-19 infections [4]. It is well established that some changes in body composition with aging are inevitable—such as the loss of bone and muscle and an increase in adipose tissue, as well as its redistribution in the body’s compartments and infiltration to other organs [5,6]. No less important is the accompanying low-grade chronic inflammation (LGCI), which is heightened with aging and the Western-type diet, thus exacerbating adverse health consequences [7].

Several years ago, the simultaneous occurrence of low bone mass (osteopenia/osteoporosis), low muscle mass/strength (sarcopenia/dynapenia), and increased/redistributed adiposity was termed osteosarcopenic adiposity syndrome (OSA) [5,8]. OSA presents the most advanced stage of body composition deterioration and includes both osteopenic/osteoporotic adiposity (OA) and sarcopenic adiposity (SA). Research throughout the world and across different ethnicities has shown many possible causes of OSA, including aging, LGCI, stress, poor nutrition, immobilization, and some chronic diseases, as reviewed recently [9,10,11,12]. Studies have also shown that the presence of OSA (aggravated with underlying LGCI) is associated with multiple functionality declines, including increased frailty and risk of fall. Additionally, metabolic deregulations in individuals with OSA (e.g., lower serum vitamin D levels, worsened serum lipid profile, and higher blood pressure) compared with other subgroups, all point to many adverse consequences of OSA [11,12] and to the need for multiple interventions—nutritional, pharmacological, and the implementation of various physical activity programs [13]. Although studies on OSA syndrome are based only on associations (no randomized clinical trials were conducted in order to implement some interventions and establish causes and effects), it is safe to assume that the OSA consequences are greater than the sum of its individual components [9,10,11,12], and the syndrome warrants future investigation.

Apart from bone, muscle, and adipose tissue deteriorations (leading to osteoporosis, sarcopenia, and obesity, respectively), body fluids also change with aging, particularly extracellular water (ECW) [14]. Recent studies have demonstrated that the ratio of ECW to total body water (TBW) is increased in people with sarcopenia and some musculoskeletal problems, and that it can be used for the early identification of individuals at risk for those diseases [14,15]. Additionally, people with obesity have an enlarged ECW compartment compared with the intracellular water (ICW) compartment because of the high ECW/ICW ratio in the adipose tissue [16,17], as well as obesity-related edema and hormonal changes related to adipose tissue [18,19]. As such, a higher ECW is used as an indicator for inflammation, while a normal ECW/TBW ratio is an indicator of body fluid balance and good nutritional status [20]. Accordingly, we hypothesize that individuals with OSA will also have an elevated ECW/TBW ratio.

These age-related changes in body composition influence nutrient metabolism and requirements in older adults [6]. A low intake of nutrient-dense foods places older adults at risk for malnutrition, especially protein and micronutrient deficiencies [11,21]. Furthermore, nutritional deficiencies, due to physiological changes (impaired mastication, swallowing, digestion, absorption, and utilization of nutrients); the presence of chronic diseases; and the influence of social and psychological factors [6,9,10,11,22] are even more pronounced in nursing homes, but are often neglected [23,24]. Nursing home residents (NHRs) are also more vulnerable to specific nutritional deficiencies because of the process of institutionalization itself, influencing food acceptance [25], eating habits, and behavior [26]. Indeed, a recent review addressed possible increased susceptibility to OSA in NHRs, based on unfavorable changes in the microbiome caused by institutionalization [27]. Because of social isolation, a lack of social support [26], and sedentary lifestyle, often commonly seen in this population, NHRs also suffer from a higher risk of depression [28]; depression being one of the main factors associated with malnutrition in this population [29]. All of this can aggravate the already reported or presumed triggers associated with OSA: reduced energy expenditure; reduced protein and omega-3 polyunsaturated fatty acids intake; insufficiencies in calcium, magnesium, potassium, and vitamins B_6_, B_9_, B_12_, A, D, E and K; and an excess of sodium and phosphorus [9,10,11,12,30,31]. Obviously, such alterations, alone or in combination, in addition to co-residency, daily interaction with staff, and congregate living, may drastically contribute to increased risk for COVID-19 infection and could result in a more serious course of the disease and a higher mortality in NHRs, as shown recently [32,33].

Based on the demographic context characterized by a growing aging population and especially by those living in nursing homes, the need for studies in this area has become extremely relevant. Presently, the need is even more intensified by the high risk for older adults, especially those living in nursing homes, to succumb to COVID-19 infections [4,32,33,34,35]. Therefore, the aim of this study was to assess the body composition (including body fluids) and dietary intake in NHRs. Furthermore, we explored the association between OSA syndrome (and its components) with specific macro- and micro-nutrients, as well as the possible influence of ECW as an indicator of LGCI. Our long-term objective is to provide more insight into the body composition and nutritional status of this population and to help ensure their good health and quality of life; a major public health priority in general, but particularly in the COVID-19 era.

## 2. Materials and Methods

### 2.1. Participants and Data Collection

All residents over 65 years of age from a nursing home in Zagreb, Croatia (total number, *n* = 355), living in the nursing home for ≥6 months were invited to participate in the study. Ethical approval was granted by the Ethics Committee of the Institute of Medical Research and Occupational Health (100–21/18–10), and the study was designed in accordance with the 1964 Helsinki Declaration and its later amendments or comparable ethical standards. The nurses from the nursing home informed the residents about the study aims and methods, and listed those individuals who were interested and were >65 years. Residents who could sit to stand independently (with or without aid) and provide informed consent were invited to participate in the study. Those who had a contradiction to bioimpedance measurement (e.g., pacemaker or amputation) and who had cancer related cachexia were not invited. The study commenced in February 2020 but had to be abruptly stopped in March due to COVID-19 pandemic lockdowns.

All measurements and interviews took place in the big hall of the nursing home with the help of nurses and other personnel. All participants were interviewed and asked about their present or past diseases (including bone fractures), prescription drugs, and smoking, as well as their dietary intake. Regarding fractures, an osteoporotic fracture was defined as either a fracture resulting from a low-impact fall in participants with a diagnosis of osteoporosis or from a fall resulting in a low-trauma fracture [36]. The number/kind of prescription drugs taken by participants at the time of the study was recorded and calculated as the “medication index”, and was included as an indicator of overall health status in some analyses.

### 2.2. Anthropometric and Bioimpedance Measurements

The participants’ body weight and height were measured with a digital scale and stadiometer (SECA 877 and 217, Hamburg, Germany) to the nearest 0.1 kg and 0.1 cm, respectively, without shoes and in indoor clothing. Body composition was assessed with a portable, advanced bioelectrical impedance device BIA-ACC (BioTekna^®^, Marcon-Venice, Italy) measuring fat-free mass (kg; % of body weight); total bone mass (kg); volumetric bone mineral density (BMD; g/cm^3^ yielding the T-score); skeletal muscle mass (kg; % of body weight; yielding the S-score); fat mass (kg; % of body weight); intramuscular adipose tissue (IMAT; as % of body weight); and ECW and ICW, as percentage of TBW, the latter being the % of body weight. The normal range of the ECW/TBW is provided by the manufacturer, and in a healthy state it should fall between 0.360 to 0.390 [37,38].

Bioimpedance measurements were carried out in the morning, with participants in a supine position with legs slightly spread and arms not touching the body. For the upper extremities, one electrode was placed on the third metacarpal area of the right hand and the second one approximately 5 cm above, centrally on the wrist area. For the lower extremities, one electrode was placed in the middle of the metatarsals area and another ~5 cm above, centrally.

Osteopenia/osteoporosis was identified based on a T-score of ≤−1.0 [12,39]. Sarcopenia was identified based on the muscle mass (with different values for women and men) yielding the S-score, with cut-off of ≤−1.0 [12]. The S-score presents the standard deviation of skeletal muscle mass with respect to healthy references individuals between 25 and 30 years old (the same approach as with T-score for bones). The total body fat cut-offs of ≥25% and ≥32% body fat for men and women, respectively, were used to determine the level of obesity/adiposity [12]. Accordingly, the simultaneous presence of osteopenia/osteoporosis and adiposity was defined as osteopenic/osteoporotic adiposity (OA), while the simultaneous presence of sarcopenia and adiposity was defined as sarcopenic adiposity (SA). Classification for osteosarcopenic adiposity included participants with simultaneous OA and SA [12]. Participants with healthy bones and muscle, but with a body fat of ≥25% for men and ≥32% for women, were classified in the adiposity-only (AD) category.

### 2.3. Dietary Intake

Dietary intake data were collected using 24-h recall. Participants indicated the type and amount of consumed food, the latter was estimated by showing kitchen dishes and utensils (cups, spoons, plates, etc.), and the manual “Quantitative models of food and meals” [40] with photographs of small, medium, and large portion sizes of individual foods/dishes. Each participant was interviewed (and helped if necessary) individually by the research staff. The official monthly menus of the nursing home were checked as well, so as to cross-reference the reported foods against the menus. Information on the type and manufacturer of the dietary supplement, as well as the daily doses consumed, were collected and added in the analysis for each nutrient estimate. The 24-h recalls were analyzed using “Nutrition” software (Infosistem d.d., Zagreb, Croatia), which contains food composition tables, as well as by utilizing the USDA data base [41] for the nutrient compositions of some foods.

### 2.4. Statistical Analysis

The results are shown as mean ± standard deviation (unless noted otherwise) for continuous variables and as percentages for categorical variables. The distribution of variables was tested using the Shapiro Wilk test. Bivariate analyses were performed depending on the type and distribution of variables. For normally distributed data (continuous variables), the independent Student’s *t*-test was used, and for non-parametric data, the Mann–Whitney U test was used to compare the differences between groups (men vs. women). The Chi-square test was used to compare the frequency of participants with osteoporotic fractures, limited mobility, smoking, and medications usage (men vs. women). ANOVA with a post hoc Scheffe test was used to analyze the differences in the anthropometric, body composition, and dietary parameters among participants stratified into groups according to body composition categories. In addition, a Pearson correlation for normally distributed continuous variables and Spearman correlation for not-normally distributed continuous variables, as well as the regression analyses, controlled for various confounders, were conducted for closer data examination. Analyses were done with IBM SPSS Statistics v. 23.0, released 2015 (IBM SPSS Statistics for Windows, Version 23.0. Armonk, NY, USA: IBM Corp.) and Statistica 13.0 software (StatSoft, Inc, Tulsa, OK, USA). The level of significance was set at *p* < 0.05.

## 3. Results

A total of *n* = 84 participants were recruited, of whom 82.2% were women (80.7 ± 6.8 years) and 17.8% men (86.3 ± 3.5 years). The average time spent in the nursing home was 4.5 and 3.7 years for women and men, respectively (Table 1). Eight women (11.6%) but no men used a walker or wheelchair. One man (6.7%) and twenty one women (30.4%) had experienced an osteoporotic fracture. Only five women and two men were current smokers. At the time of the study, 82.0% of women and 66.6% of men were taking antihypertensive medications, and 13% of women and men were taking medications for hyperlipidemia. Only two women were on diabetes medication and another two on osteoporosis medication. The medication index was not significantly different in women (3.1 ± 4.8) and men (2.7 ± 2.9; Table 1).

Descriptive statistics of the anthropometric and body composition parameters presented in Table 1 showed that men, compared with women, had significantly higher body height, fat free mass, bone mass, and muscle mass (all expected). Both women and men were overweight according to the body mass index (BMI) and the amount of body fat; however, women had significantly higher BMIs and body fat compared with men. Men had a significantly higher percentage of TBW and ICW distribution, as well as lower ECW (as % of TBW) compared with women (Table 1).

Regarding dietary intake, women and men had a similar energy, macronutrient, and micronutrient intake (Table 2 and Table 3). Based on the European Food Safety Authority (EFSA) recommendations, both women and men had a lower than recommended protein, omega-3 fatty acids, and fiber intake and a higher saturated fatty acids intake. All participants had an adequate intake of only vitamins B_1_ and B_3_, phosphorus, and selenium; in addition, women had an adequate intake of vitamins B_2_, B_6_, and C (probably due to the supplements). The intake of all other vitamins and minerals was below the EFSA recommendation for both women and men (Table 3). This low intake was despite the fact that 49.3% of women (*n* = 34) were taking vitamin and/or mineral supplements (included in the total estimate presented), of which the most commonly taken were vitamins B_1_ (17.7%), B_2_ (20.6%), B_3_ (23.5%), B_6_ (52.9%), and C (50.0%), as well as minerals calcium (17.7%), magnesium (76.5%), and potassium (12.8%). Two men were taking calcium supplements only.

**Figure 1:**
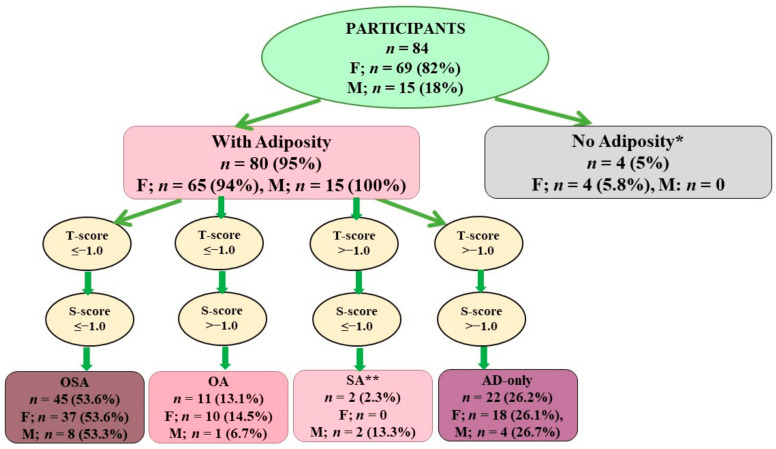
Classification of participants according to body composition compartments. * Participants had both osteoporosis and sarcopenia, but because of a small number they were not analyzed further. ** Participant with only SA were not analyzed further because of a small number. Different criteria are used for determination of osteopenia/osteoporosis, sarcopenia, and adiposity for women and men. F—female; M—male; OSA—osteosarcopenic adiposity; OA—osteopenic/osteoporotic adiposity; SA—sarcopenic adiposity; AD—adiposity only.

The classification of participants into body composition categories is presented in Figure 1. The participants without osteopenia/osteoporosis and sarcopenia, but with adiposity, were classified in the AD-only (adiposity-only; 26.2%) group, those with osteopenia/osteoporosis and adiposity in the osteopenic/osteoporotic adiposity (OA; 13.1%) group, and those with all three conditions in the osteosarcopenic adiposity (OSA; 53.6%) group. There were only 5% (*n* = 4, all women) of participants without adiposity and 2.3% (*n* = 2, all men) with sarcopenic adiposity. They were excluded from the further analyses because of the small numbers (Figure 1).

**Figure 2:**
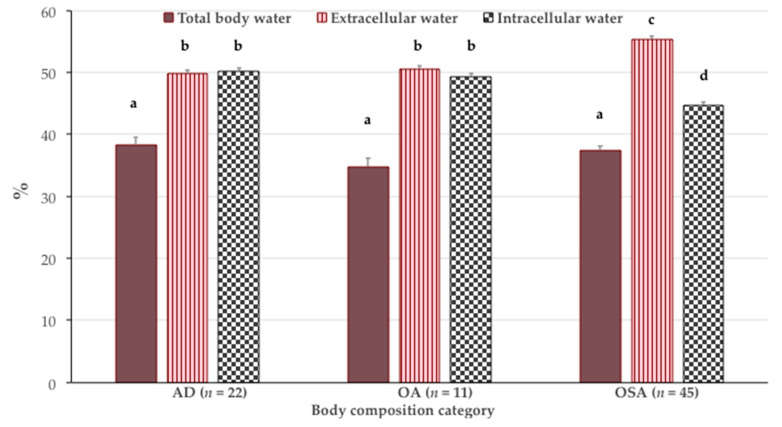
Percent of total body water (TBW), extracellular water (as % of TBW) and intracellular water (as % TBW) in participants according to body composition categories (mean ± standard error). Bars with different letters indicate the statistically significant difference among the groups (ANOVA with a post hoc Scheffe test; *p* < 0.05). AD—adiposity only; OA—osteopenic/osteoporotic adiposity; OSA—osteosarcopenic adiposity.

Table 4 presents the anthropometric and body composition parameters of the participants stratified by body composition categories. The age, height, and TBW% (Figure 2 for the latter) did not differ among the groups, while body weight differed significantly for all three groups, with AD participants having the highest weight and OSA participants the lowest weight. While the OSA participants had a significantly higher fat free mass, the AD participants had a significantly higher bone mass, and, accordingly, the T-score differed between the groups, with the OSA participants having the lowest values. Similarly, the AD participants had the highest S-score and percentage of muscle mass, followed by those in the OA and OSA groups. The OSA participants had a lower fat mass and percentage of intramuscular adipose tissue compared with those in the OA and AD groups (Table 4).

Figure 2 depicts the distribution of body fluids. Although the participants in all three groups had a similar content of TBW, participants with OSA had a significantly higher distribution of ECW (55.3 ± 4.2% of TBW; *p* < 0.001) and lower ICW (44.7 ± 4.2% of TBW; *p* < 0.001) compared with the participants in AD (ECW 49.8 ± 2.2% of TBW and ICW 50.2 ± 2.2% of TBW) and OA (ECW 50.6 ± 1.6% of TBW and ICW 49.4 ± 1.6% of TBW), the latter two having a similar distribution (Figure 2).

In terms of dietary intake (Table 5 and Table 6), there was no significant difference in energy and macronutrient intake among the groups, except for protein intake (% kcal), which was highest in the AD group. However, all participants had protein, omega-3 fatty acids, and fiber below and saturated fatty acids above the recommendations. The micronutrient intake was also similar among participants in all three groups, with only selenium intake being significantly higher in the AD group compared with OA and OSA groups. Most of the micronutrients were below the recommendations, despite the vitamin/mineral supplement intake among women.

**Figure 3:**
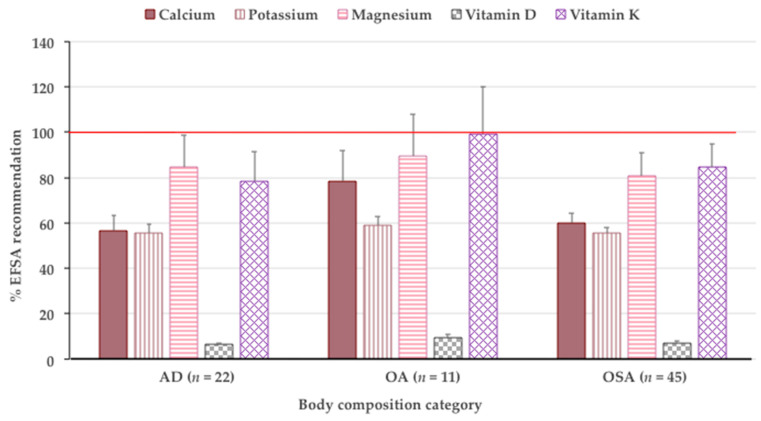
Select minerals and vitamins in reference to EFSA recommendations in participants according to body composition categories (mean ± standard error). ANOVA is not significant. Horizontal red line denotes 100% compliance with recommendations. EFSA—European Food Safety Authority; AD—adiposity only; OA—osteopenic/osteoporotic adiposity; OSA—osteosarcopenic adiposity.

Figure 3 presents the % of intake of selected micronutrients in relation to the EFSA recommendations. The nutrients selected are those recently reported as having the highest influence on body composition [11]. There was no significant intergroup difference in compliance with the EFSA recommendations for calcium, potassium, magnesium, vitamin D, and vitamin K intake (Figure 3). Participants in all three groups did not meet the EFSA recommendation for any of the selected vitamins and minerals, except those in OA group, who barely reached the recommendations for vitamin K.

In the correlational analyses, only a few nutrients showed statistical significance with body composition components (not presented). For example, in both the AD and OA groups, protein intake was positively related to ICW and bone mass and negatively to ECW, while fat intake was negatively related to bone mass. Additionally, in the OA group, fiber intake was positively related to muscle and bone mass, and vitamins A and K were positively to bone mass. In the OSA group, protein intake was positively related to the T-score, and carbohydrate intake to BMI, body fat, and intramuscular fat (all *p* < 0.05). In the logistic regression model (controlled for age), with OSA as a dependent variable and ECW, protein intake, years in residency, and medication index as predictors, significance was reached for ECW and years in residency. The odds ratio for ECW was 1.5 (CI: 0.3–1.7, *p* < 0.001), while the odds ratio for years in residency was 1.1 (CI: 1.0–1.2, *p* = 0.01).

## 4. Discussion

To our knowledge, this is the first study to report on the prevalence of OSA (and its components) and its association with dietary intake in older adults living in nursing homes, as well as to examine the possible influence of ECW (indicator of inflammation) in relation to body composition. Our results showed a high prevalence of OSA (>53%) in both women and men (Figure 1), with ECW% having the strongest positive relation with the OSA group compared with the OA and AD groups of participants (Figure 2), as well as being significant in the logistic model, along with years in residency (results not presented). The analysis of diet revealed a poor dietary intake of most nutrients being below the recommended levels, but particularly those related to OSA and some to increased risk of COVID-19 infections, namely, protein; omega-3 fatty acids; calcium; magnesium; potassium; vitamins C (in men), D, E, and K; and zinc (Table 2 and Table 3 and Figure 3). However, there was no significant difference in dietary intake among the participants in different groups (OSA compared with OA or to AD). These findings confirm some reasons and point to others about why NHRs might potentially be at such high risk of COVID-19 infection and the subsequent detrimental outcomes, as reported in the literature [32,33].

As aging uniquely influences many physiological functions, the most observable are those regarding body composition changes, including loss of bone, loss of muscle mass and strength, and increased and/or redistributed adiposity, all potentially leading to the most advanced deterioration, known as OSA syndrome [6,8]. Presently, there is still no consensus for OSA classification–diagnostic criteria, mostly due to the disagreement about adiposity cut-offs, as well as the variety of populations in which these studies have been conducted [12]. Therefore, it is hard to estimate the overall prevalence of OSA [11,12]. Furthermore, there are no data on the prevalence of OSA in nursing homes. Our findings on OSA prevalence (reported for the first time in this study) and detecting 53.6% and 53.3% of women and men with OSA, respectively (measured by convenient, portable, and noninvasive BIA technologies), were alarming. Some other studies in older adults showed a prevalence of OSA ranging from 10 to 30%, as reviewed recently [11,12]. For example, a Turkish study investigated community-dwelling individuals >65 years and found a 10.7% prevalence of OSA [42]. Considering that our participants were on average much older (80.7 and 86.3 years old for women and men, respectively) and living in nursing homes, the latter known to adversely affect both body composition and nutrition [9,10,11,12,30,31,32,33,34,35], the prevalence of OSA was expected to be higher.

Reports from some studies indicate that a higher ECW/TBW ratio can be predictive of sarcopenia [14,15]. During aging, TBW decreases, mostly because of a decrease in ICW [43], because of a reduction in muscle mass relative to fat mass [44]. As more than 70% of muscle tissue consists of water, it is expected that water loss disturbs muscle function. An age-related decrease in ICW may reflect a reduction in muscle cell hydration, which may have implications on the mechanical and metabolic functions of muscle [45]. As the ECW/TBW ratio reflects the movement of water from the intracellular to extracellular compartment, along with changes in the cell membrane potential, it could be used as an indicator of the water balance within the body to mark the edema, the result of adipose tissue expansion [15,16], or the early detection of sarcopenia [45]. Other studies have reported that individuals with sarcopenia have a higher ECW% compared with those without sarcopenia [14,46,47]. Our results show that ECW% was the highest in the participants with OSA compared with those with OA and AD, again uniquely indicating the most impaired state in the presence of OSA syndrome.

The issue of body fluids also attracts attention in the field of obesity and nutrition, as a high ECW/ICW is noted in obese individuals due to obesity-related edema and the hormonal responses of adipose tissue itself [17]. It has also been shown that obese women have a lower TBW per unit of weight and a higher ECW per unit of TBW compared with normal-weight women [48]. Therefore, it is reasonable to conclude that the higher adipose tissue in our participants resulted in the observed higher ECW, particularly in the OSA participants with concomitant deteriorated bone and muscle tissue. Furthermore, age-related changes in protein metabolism, especially in muscle, and a slower utilization of dietary protein, both contribute to the changes within the fat-free mass in a way that intracellular mass is depleted relative to extracellular mass [49]. Age-related changes and interrelationships in the metabolism of bone, muscle, and adipose tissue [5,6] indicate that ECW% may be an indicator not only of sarcopenia, but also of OSA, a unique finding of our study.

The analysis of dietary intake revealed no significant difference in energy and macronutrient intake between participants with OSA, OA, and AD, except for protein (% kcal), which was highest in the AD group, despite that it was below the recommendations for all groups (Table 5). A similar situation was observed with micronutrients, among which only selenium intake was significantly higher in the AD group (Table 6). A probable reason for no difference in intake among the groups is because NHRs consume their meals primarily in nursing home dining halls and rarely eat outside; therefore, the quality of their diet is the same, with just small individual differences in the quantity. Some results from previous studies regarding energy and macronutrient intake in community-dwelling individuals are similar to ours, with no difference in intake in postmenopausal women among those with OSA compared with those with fewer abnormalities (OA, SA, and AD) [50,51]. On the contrary, in studies by Kim et al. [52,53], older (average 61 years) community-dwelling adults with OSA had a significantly lower energy intake compared with those with only one or two OSA components. In a study by Choi et al. [54], a lower energy intake was found only in male participants with OSA compared with participants with fewer OSA components.

Protein intake, expressed as g/day, was significantly lower in both women and men with OSA compared with those in other body composition categories in the studies by Kim et al. [52,53], but there was no difference in protein intake in a study by Choi et al. [54]. Additionally, other studies [50,51] have reported no difference in protein intake in postmenopausal women stratified by different body composition abnormalities, and the same was true with carbohydrate and fat intake (g/day) [50]. All of these studies were conducted in free-living participants. Based on the above, and corroborated by the results of our study, it is still hard to reach a consensus about the association of macronutrients and OSA, except possibly for protein. Although still not unanimously confirmed, it seems that a lower protein intake is related to a higher prevalence of OSA, particularly in women.

A few studies have reported on the intake of micronutrients in participants with OSA and compared it with participants with milder body composition abnormalities (e.g., OA, SA, or AD) [50,51,53]. Accordingly, only male participants with OSA had a lower calcium intake compared with those with fewer body composition abnormalities, while female participants had a similar intake [53]. In a study among postmenopausal women (aged 50–64 years), those with OSA had a lower vitamin C and potassium intake (possibly due to lower fruit intake) compared with women with fewer body composition abnormalities, while the intake of vitamin A, B_1_, B_2_, and B_3_; calcium; phosphorus; sodium; and iron was similar in participants from all groups [50]. The intake of vitamins C and E was lower in the OSA group, while calcium intake was similar among the groups in another study conducted in postmenopausal women, where the dietary inflammatory index was measured [51]. Our results show that participants with AD, OA, and OSA did not meet the EFSA recommendations for most of the micronutrients, particularly calcium, magnesium, potassium, and vitamins D and K (Figure 3). These minerals and vitamins are particularly emphasized, because a recent review highlighted the importance of their adequate intake concerning their metabolic and physiological roles in relation to OSA and LGCI [11]. In addition, our participants did not meet the vitamin C (men only because the women were taking supplements) and zinc recommended intake—both antioxidants and/or involved in immune system functioning, thus likely being protective toward COVID-19 infections.

The reason for the reported discrepancy in the literature for energy and nutrient intake could be explained by the use of different methodologies to assess dietary intake and the inherent limitations in any of the dietary surveys, well recognized and discussed previously [55], which depends on the memory and objectivity of the respondents. Furthermore, the studies were performed in participants of different sex, age, ethnicity, and sociodemographic characteristics, while adiposity and sarcopenia were determined with different methods and using different cut-offs [12]. This all indicates the need for more studies in this area.

Besides the poor body composition and high prevalence of OSA, our study revealed reasons for concern regarding inadequate nutrition among NHRs, especially considering that long-term inadequate nutrient intake may promulgate the occurrence of OSA [30,31], as well as increase the risk for COVID-19 infections even more [9,10,11,12]. The recommendation for energy intake in older adults is 25–30 kcal/kg/day, which would range from 1750 to 2100 kcal/day for a normal-weight older individuals [12,56], and the EFSA recommendation ranges from 1625 to 2844 kcal/day [57], depending on the age, sex, and the level of physical activity. For individuals over 80 years old, the energy intake recommendation has not been determined [57]. The average energy intake in our participants was lower than both the Dietary Reference Intakes (DRI) [56] and EFSA [57] recommendations (Table 2 and Table 5), which sounds paradoxical since most of them were overweight. However, the existing discrepancy between energy intake and weight/obesity has been reported in other studies and is frequently discussed [6]. Consistent with our study, energy intake (1607.8 ± 396.0 kcal) was below the recommended level for the elderly NHRs in France [58]. However, a German study showed that elderly NHRs had a higher energy intake than the participants of our study (e.g., 2017 ± 528 kcal and 1731 ± 451 kcal in men and women, respectively) [59].

An interesting study was performed in one Belgian nursing home, where the energy and protein content of the served food was compared with the actual food consumed by NHRs, reporting that residents consumed just fragments of the meals, and the actual energy and protein (1552.4 ± 342.1 kcal and 0.88 ± 0.25 g/kg/day, respectively) content of the consumed food was considerably less than what was served [60]. Despite the lower consumption, the energy and protein intake in that population was still higher than in our participants, although we did not compare the served vs. consumed food in our study. Similarly, a study performed in Australian residential care facilities highlighted that a mean energy of 1953 kcal/day was served to the residents, but the mean energy consumed was 1576 kcal [61]. This low intake is most likely due to several reasons, as addressed in the Introduction and discussed in earlier literature [6,62,63].

Overall, it seems that an insufficient energy and protein intake is common among residents of nursing homes [58,60,63], which could cause higher than normal bone and muscle loss; increase frailty and susceptibility to other diseases [9]; and, in the time of the COVID-19 pandemic, increase infections and worsen prognosis [9,10,11,12]. It is well established that low-protein diets (even at the level of 0.7 g/kg) interfere with normal bone metabolism, calcium absorption, and the synthesis of growth factors, compromising both bone and muscle [64,65]. The current suggestions are to achieve approximately 25% of the total energy intake in the form of a good-quality protein, for which meat, poultry, fish, soy, dairy, and eggs are the best sources, as they have the adequate amounts of all essential amino acids [10,12]. According to some studies, protein intake for older adults should be about 1.0–1.2 g/kg/day [12,66], which is higher than the current DRI recommendation of 0.8 g/kg/day [56] or EFSA recommendation of 0.83 g/kg/day [57] for all adults. The protein intake of our study participants was about 0.7 g/kg/day and about 15% of energy (Table 2 and Table 5), which is below the recommendations for optimal maintenance of body composition by both European and US recommendations [56,57]. A similar intake was also reported for elderly NHRs in France [58].

Dietary fat intake should amount to 20–35% of energy intake, and saturated fatty acid should be as low as possible [57] or below 7% of the energy intake [56]. While the cholesterol intake of our participants was within and the fat intake was at the higher end of recommendations, the saturated fatty acid intake was almost double in reference to recommendations (Table 2 and Table 5). The recommendations for fat intake are focused on increasing polyunsaturated fatty acids, particularly omega-3 fatty acids, which have anti-inflammatory and anti-obesity effects, and also provide benefits for bone, muscle, and adipose tissue health [7]. Ideally, a diet should include approximately 1 g/day of omega-3 fatty acids [67]. While it is widely recognized that fatty fish (e.g., salmon, herring, tuna, and sardines) are the best sources of omega-3 fatty acids, some plant foods (walnuts, flaxseeds, and chia seeds) also provide good amounts. The average intake of omega-3 fatty acids was very low in our study participants (about 10% of recommendations), reflecting the scarcity of omega-3 fatty acid-rich foods offered on the daily menu (see discussion below). However, the average carbohydrate intake in our study participants was in accordance with recommendations (45–60% kcal/day) [57], but average fiber intake was below recommendation of 25 g/day [57]. The recommended amounts of the latter are harder to achieve in older adults with a generally lower food intake, but particularly in those with a scarce intake of fresh fruits and vegetables.

In terms of micronutrients, most of the minerals (calcium, magnesium, potassium, iron, and zinc) were below recommendations, phosphorus was above recommendations, while sodium was in accordance with recommendations [57] for both women and men (Table 3 and Table 6). About 49% of women were taking vitamin supplements that increased their average intake of vitamins B_1_, B_2_, B_3_, B_6_ and C above recommendations. Men were below recommendations for all vitamins, except for vitamins B_1_ and B_3_. Of note is the extremely low intake of vitamin D in all participants (about 6% of recommendations [57], Figure 3). The deficiencies of vitamins such as B, C, and D and minerals such as calcium, magnesium, and zinc have been reported in other studies among Brazilian nursing home residents [68,69], particularly when their intake was estimated only from food (without the addition of dietary supplements). Similarly, in a recent study conducted in Canadian long-term care residents, the intake of several micronutrients was inadequate for at least 50% of participants of both sexes [70]. These nutrients included vitamins D, E, K, B_6_, and B_9_; calcium; magnesium; and potassium, which is in line with the findings of our study.

Vitamin A intake in our participants was also below recommendations. It appears that both a too high and too low intake of vitamin A could impair bone health in older individuals [64,71]; therefore, it is important to follow recommendations, particularly in view of the popularity of vitamin A supplements in older women [64]. The impact of B vitamins on bones [72] and muscle is inconclusive or non-existent [70], and has been discussed recently [9,73]. Vitamin C is required for collagen crosslinking; thus, its low intake or deficiency could lead to a weakening of the collagenous structure in bone and to other impairments related to a low antioxidant intake [9,10,11,12,64]. In a recent study, Lewis et al. reported a positive correlation between dietary and circulating levels of vitamin C and muscle mass in older men and women [74]. As vitamin C is a strong antioxidant and is involved in immune system regulation, its inadequate intake may lead to an increased risk of infections, particularly relevant in the COVID-19 era [10].

Vitamin D has received a lot of attention recently, not just for its undisputed role in bone metabolism and muscle functioning, but also as a potential anti-inflammatory and immunity-enhancing nutrient [11]. Higher serum levels of 25-hydroxyvitamin D are associated with improved balance, lower fall risk, and increased muscle performance, while its deficiency may be associated with sarcopenia and numerous other disorders [75]. Indeed, several studies have reported worsened outcomes of COVID-19 infection and poorer viral defense in patients with low serum 25-hydroxyvitamin D [76,77]. The average intake of vitamin D for our participants was very low (about 6% of recommendations [57], Figure 3, presumably due to the lack of food rich in vitamin D (oily fish—salmon, mackerel, and sardines) on the menu in nursing homes. Regarding vitamin E, a study by Welch et al. reported that a higher intake of vitamin E was associated with better lean tissue indices (as a proxy of muscle mass), in combination with vitamin C and carotenoids [78]. High plasma levels of vitamin K were associated with muscle strength, larger muscle mass, and higher physical performance in some observational studies [79]. Its role in bone for the carboxylation of osteocalcin is well established and has been recently discussed [11]. However, the intake of both vitamin E and K in our study participants was also far below the recommendations, thus placing them all at higher risk of OSA.

Calcium and magnesium are well known for their role in bone, muscle, and adipose tissue health [64,80], and they will not be discussed further here. However, an interesting recent study conducted in Croatian nursing homes reported a low dietary calcium intake (528 ± 279 mg/day women and 653 ± 296 mg/day, men) in 292 older (>80 years) participants [81]. This intake is very similar to the intake of our participants, except that in the study of Cvijetić et al., the daily calcium intake was significantly higher in men than in women, although neither of them reached the recommended levels [57].

As around 90% of zinc in the human body is located in muscle, bone, skin, and hair, it is obvious that a zinc deficiency may result in impaired bone formation, as well as in muscle and connective tissue structure and metabolism disturbances, as reviewed earlier [64]. In a Korean study [82], the serum zinc concentration was positively associated with both abdominal obesity and total body fat in adult men, although there was no relationship between serum zinc concentrations and body composition components in women [82]. Zinc, another nutrient crucial for immune system functioning and viral defense, intake in our participants was also below the recommendations. In a recent study conducted in COVID-19 patients, serum zinc levels were low and linked to higher mortality rates, even after adjusting for relevant confounders [83]. Phosphorus is a major component of bone crystal—hydroxyapatite—and it is crucial for muscle functioning as part of creatine [10]. However, because of its abundance in all foods and its widespread addition to soft drinks and processed foods, its intake is typically above the recommendations in Western societies. In our study, the average phosphorus intake was above the recommendations in both sexes and in all body composition categories; however, it did not reach high levels as seen in some other studies [80], and was low enough to not cause concern regarding its possible negative side effects.

Considering the relatively long-time residency in nursing homes (4.5 and 3.7 years on average for women and men respectively), and the multiple comorbidities and medications in NHRs (Table 1), a healthy diet with adequate amounts of both macro- and micro-nutrients would be of crucial importance. The menus in the nursing homes in Croatia are very similar and are rotated on a monthly basis. Milk and/or tea and bread topped with butter, margarine, or some other spread are offered every morning. Meat or meat dishes are offered every day except Friday, and fish is offered only 2–3 times per month. Fresh vegetables are offered only as a salad, while other cooked vegetables may be offered as part of a soup or another cooked dish. Fruits are offered 9–10 times per month, but sometimes either canned or cooked. Yogurt, rice milk, or pieces of cheese are regularly offered as snacks. Although fashioned appropriately, the overall menu lacks in fish, fresh fruits and vegetables, whole grains, and nuts and seeds, and is unable to provide adequate nutrition to the residents, especially if some of them do not consume all of the offered food. Therefore, special attention should be paid to the diet and nutritional supplements offered in order to avoid nutritional deficiencies and keep residents healthy and resistant to infectious and other chronic diseases, especially during the COVID-19 pandemic.

The limitations of our study are its cross-sectional nature, its relatively small sample size, and the unequal number of women and men, although this is not uncommon in other studies. The small sample size might have particularly been a case in subgroup analyses in which the number of participants was even more reduced and thus a statistical power could not be reached for possible significance. On the other hand, in a smaller study, it is possible to recruit a more uniform sample with strict exclusion/inclusion criteria and to measure an array of important biological and lifestyle variables, and to account for them as potential confounders. Even more so, it is possible to collect subject-based recalls and self-reported data more accurately, as participants could be individually instructed and interact with researchers, as was the case in our study.

Another limitation is the dietary assessment via 24-h dietary recall, which, as with any other dietary record, has inherent shortcomings [55]. However, this seems to be the most applicable way of gathering dietary information in a setting where participants are not under strict monitoring (e.g., in a metabolic ward). Moreover, the menu in the nursing home is predetermined and known, thus there is less variety and unpredictability in participants’ intake.

We did not collect the data on the physical activity of NHRs. However, as a recent study in almost 300 NHRs in Zagreb reported [84], all nursing homes in Croatia do have organized exercise programs for a duration of 30 min every morning, which include different exercises and stretching in sitting and standing positions. However, the frequency and level of involvement of NHRs in such kinds of activities is questionable, and presents another important factor to assess in their physical fitness and overall health. As was recently reviewed [85], exercise programs and especially movement-based interventions could be useful in rehabilitation and for the maintenance of overall fitness. Similarly, Ghram et al. addressed the importance of home-based exercise for older adults in general, but particularly during the pandemic when no other activities or movements are available/possible [86]. Physical activities and exercise are a very important topic; however, they are beyond the scope of this paper, as physical activity in our participants was not assessed.

Our study began in February 2020 just before the COVID-19 pandemic caused massive lockdowns and the closure of nursing homes to visitors. Therefore, we present these results as a pilot study with the aim to continue and build upon it as soon as the restriction measures are lifted. In view of the COVID-19 pandemic and the recognized higher risk of infection among NHRs [32,33], we believe that these results contribute to the awareness and importance of body composition and diet in nursing home settings. OSA and its components among elderly people in nursing homes, accompanied with poor diet and inadequate nutrition, could be a serious problem and could elevate the risk of infection. Obviously, maintaining physical abilities in old age is an important factor and along with standard medical care, the optimization of body composition and nutritional status should be included for the prevention of OSA and malnutrition in order to enable better resistance in the COVID-19 era.

## 5. Conclusions

Our results indicate the high prevalence of OSA among NHR and significantly elevated ECW in OSA participants compared with those with other body composition impairments, indicating a higher inflammatory state for the former. The analysis of dietary intake showed no significant difference in energy, macronutrient, and micronutrient intake between participants with OSA, OA, and AD, except for protein intake (% kcal) and selenium. However, all participants had a poor dietary intake, with most of the nutrients below the recommended levels, including protein, omega-3 fatty acids, and fiber, as well as most of micronutrients, particularly vitamin D. Providing adequate and nutritious food to older adults in nursing homes is crucial to ensure nutritional adequacy, optimal body composition, and to maintain their health and a good quality of life. In order to avoid a deficiency of macro- and micro-nutrients in NHRs, possible dietary supplements should be considered.

## Figures and Tables

**Table 1:** Characteristics of participants with body composition parameters ^1^.

Parameters	Women (*n* = 69)	Men (*n* = 15)	*p* Value *
Age (years)	80.7 ± 6.8	86.3 ± 3.5	<0.001
Years in residency (years)	4.5 ± 3.7	3.7 ± 3.5	0.499
Osteoporotic fractures (n)	21 (30.4%)	1 (6.7%)	0.058
Limited mobility (n)	8 (11.6%)	0	/
Smoking (n)	5 (7.2%)	2 (2.8%)	0.528
Medications for:			
Hypertension (n)	57 (82.0%)	10 (66.6%)	0.193
Hyperlipidemia (n)	9 (13.0%)	2 (13.3%)	0.975
Diabetes (n)	2 (2.8%)	0	/
Stroke prevention (n)	5 (7.2%)	2 (13.3%)	0.437
Osteoporosis (n)	2 (2.8%)	0	/
Hypothyroidism (n)	4 (5.7%)	0	/
Medication index	3.1 ± 4.8	2.7 ± 2.9	0.757
Body Composition			
Height (cm)	156.1 ± 6.1	170.6 ± 6.7	<0.001
Weight (kg)	72.3 ± 14.2	79.3 ± 11.2	0.078
Body mass index (kg/m^2^)	29.6 ± 5.2	27.2 ± 3.2	0.027
Fat free mass (% body weight)	57.8 ± 5.9	63.3 ± 5.4	0.001
Total bone (kg)	2.7 ± 0.6	3.8 ± 0.6	<0.001
T-score	−1.4 ± 0.9	−1.0 ± 0.6	0.099
Muscle mass (% body weight)	14.9 ± 2.1	21.8 ± 2.1	<0.001
S-score	−1.2 ± 1.5	−1.3 ± 0.9	0.853
Fat mass (% body weight)	42.2 ± 5.9	36.7 ± 5.4	0.001
Intramuscular adipose tissue (% body weight)	3.0 ± 0.3	2.9 ± 0.3	0.268
Total body water (% body weight)	36.4 ± 3.2	45.1 ± 5.0	<0.001
Extracellular water (% TBW)	54.7 ± 5.3	49.1 ± 2.7	<0.001
Intracellular water (% TBW)	45.3 ± 5.3	50.9 ± 2.7	<0.001

^1^ All continuous variables are presented as mean ± standard deviation and categorical variables as frequency (percentages). * Group differences were tested using Student’s *t*-test for parametric, and Chi-square test and Mann-Whitney U test for non-parametric variables. TBW = total body water.

**Table 2:** Average daily energy and macronutrient intake in participants ^1^.

Parameters	Women (*n* = 69)	Men (*n* = 15)	*p* Value *
Energy (kcal)	1363.3 ± 330.0	1364.8 ± 425.9	0.987
Total protein (g)	50.5 ± 14.4	55.1 ± 20.3	0.304
Total protein (g/kg)	0.7 ± 0.2	0.7 ± 0.2	0.594
Total protein (% kcal)	15.0 ± 3.3	16.3 ± 4.1	0.208
Total fat (g)	54.8 ± 21.6	48.3 ± 17.1	0.277
Total fat (% kcal)	35.3 ± 9.1	32.0 ± 6.8	0.189
Saturated fatty acids (g)	23.1 ± 10.3	20.6 ± 6.8	0.307
Saturated fatty acids (% kcal)	14.8 ± 4.8	13.8 ± 3.1	0.301
Omega–3 fatty acids (g)	0.1 ± 0.6	0.1 ± 0.1	0.944
Cholesterol (mg)	135.8 ± 99.6	149.5 ± 55.2	0.187
Carbohydrate (g)	172.7 ± 43.1	185.4 ± 65.2	0.351
Carbohydrate (% kcal)	51.4 ± 9.6	54.1 ± 7.5	0.316
Dietary fiber (g)	15.9 ± 5.8	17.5 ± 7.2	0.335

^1^ All of the variables are presented as mean ± standard deviation. * Group differences are tested using Student’s *t*-test for parametric variables and Mann–Whitney U test for non-parametric variables.

**Table 3:** Average daily micronutrient intake, including food and supplements, and the corresponding EFSA recommendations ^1^.

Parameters	Women (*n* = 69)		Men (*n* = 15)		*p* Value *
	Intake /Day	EFSA	Intake/Day	EFSA	
Vitamins					
Vitamin A (µg RE) ^2^	421.2 ± 269.6	650	419.7 ± 254.4	750	0.986
Vitamin B_1_ (mg/MJ) ^3^	0.5 ± 1.4	0.1	0.1 ± 0.1	0.1	0.875
Vitamin B_2_ (mg)	3.3 ± 8.8	1.6	0.8 ± 0.3	1.6	0.939
Vitamin B_3_ (mg NE/MJ) ^4^	2.5 ± 2.0	1.6	2.2 ± 0.8	1.6	0.362
Vitamin B_6_ (mg)	3.5 ± 8.0	1.6	1.0 ± 0.5	1.7	0.158
Vitamin C (mg)	167.6 ± 178.8	95	82.3 ± 57.7	110	0.038
Vitamin D (µg)	1.1 ± 0.7	15	0.1 ± 0.4	15	0.730
Vitamin E (mg)	5.4 ± 2.1	11	4.5 ± 2.4	13	0.172
Vitamin K (µg)	58.9 ± 46.4	70	58.0 ± 53.9	70	0.766
**Minerals**					
Calcium (mg)	576.3 ± 285.0	950	638.4 ± 397.4	950	0.949
Copper (mg)	1.0 ± 0.4	1.3	1.0 ± 0.6	1.6	0.856
Iron (mg)	7.4 ± 2.4	11	7.4 ± 2.1	11	0.977
Magnesium (mg)	275.8 ± 206.8	300	135.3 ± 58.5	350	0.083
Phosphorus (mg)	776.4 ± 229.5	550	841.4 ± 289.1	550	0.378
Potassium (mg)	1941.9 ± 553.7	3500	1967.5 ± 614.4	3500	0.866
Selenium (µg)	72.3 ± 20.8	70	80.7 ± 32.3	70	0.204
Sodium (mg)	1587.1 ± 676.4	2000	1526.5 ± 688.2	2000	0.875
Zinc (mg)	5.6 ± 2.6	7.5	6.5 ± 2.9	9.4	0.185

^1^ All of the variables are presented as mean ± standard deviation. * Group differences are tested using Student’s *t*-test for parametric variables and Mann–Whitney U test for non-parametric variables. EFSA—European Food Safety Authority; ^2^ RE—retinol equivalent (1 µg RE = 1 µg of retinol); ^3^ The thiamin requirement is related to the energy requirement, and is therefore expressed in mg/MJ; ^4^ NE—niacin equivalent (1 NE = 1 mg niacin; niacin requirement is related to energy requirement and is therefore expressed in mg NE/MJ); MJ—megajoule.

**Table 4:** Anthropometric and body composition parameters of participants according to body composition categories ^1^.

Parameters	AD (*n* = 22)	OA (*n* = 11)	OSA (*n* = 45)	*p* Value *
Age (years)	81.7 ± 6.3	78.0 ± 7.8	82.4 ± 6.7	0.165
Height (cm)	161.6 ± 6.9	155.6 ± 4.9	158.1 ± 8.4	0.079
Weight (kg)	88.0 ± 1.6 ^a^	79.8 ± 7.5 ^b^	67.5 ± 9.2 ^c^	<0.001
Body mass index (kg/m^2^)	33.8 ± 4.0 ^a^	33.0 ± 3.4 ^a^	27.0 ± 2.8 ^b^	<0.001
Fat free mass (% body weight)	55.8 ± 4.8 ^a^	53.6 ± 5.1 ^a^	59.8 ± 4.3 ^b^	<0.001
Total bone (kg)	3.7 ± 0.6 ^a^	3.0 ± 0.3 ^b^	2.6 ± 0.5 ^b^	<0.001
T-score	−0.2 ± 0.6 ^a^	−1.2 ± 0.1 ^b^	−1.8 ± 0.5 ^c^	<0.001
Muscle (% body weight)	18.4 ± 2.9 ^a^	15.9 ± 2.4 ^b^	15.3 ± 2.8 ^c^	<0.001
S-score	0.5 ± 1.0 ^a^	−0.5 ± 0.4 ^b^	−2.0 ± 0.7 ^c^	<0.001
Fat mass (% body weight)	44.2 ± 4.8 ^a^	46.4 ± 5.1 ^a^	40.2 ± 4.3 ^b^	<0.001
Intramuscular adipose tissue (% body weight)	3.0 ± 0.2 ^a^	3.1 ± 0.3 ^b^	2.9 ± 0.2 ^c^	0.023

^1^ All of the variables are presented as mean ± standard deviation. * Intergroup differences are tested using ANOVA with a post hoc Scheffe test (*p* < 0.05). ^a,b,c^ Different superscripts indicate statistical difference among groups. AD—adiposity only; OA—osteopenic/osteoporotic adiposity; OSA = osteosarcopenic adiposity.

**Table 5:** Average daily energy and macronutrient intake according to body composition categories ^1^.

Parameters	AD (*n* = 22)	OA (*n* = 11)	OSA (*n* = 45)	*p* Value *
Energy (kcal)	1353.1 ± 311.5	1443.5 ± 440.4	1376.9 ± 337.9	0.777
Total protein (g)	57.0 ± 15.5	52.3 ± 14.9	49.7 ± 14.7	0.177
Total protein (g/kg)	0.6 ± 0.2	0.7 ± 0.2	0.7 ± 0.2	0.172
Total protein (% kcal)	17.0 ± 3.6 ^a^	14.7 ± 3.0 ^b^	14.6 ± 3.3 ^b^	0.021
Total fat (g)	53.4 ± 20.0	59.8 ± 27.3	53.5 ± 20.4	0.661
Total fat (% kcal)	35.1 ± 9.1	36.0 ± 7.0	34.2 ± 9.0	0.803
Saturated fatty acids (g)	22.1 ± 8.5	25.0 ± 11.3	22.8 ± 10.2	0.716
Saturated fatty acids (% kcal)	14.4 ± 3.8	15.3 ± 3.7	14.5 ± 5.0	0.833
Omega—3 fatty acids (g)	0.1 ± 0.03	0.1 ± 0.1	0.2 ± 0.7	0.555
Cholesterol (mg)	124.5 ± 108.6	171.9 ± 145.5	136.3 ± 69.8	0.397
Carbohydrate (g)	166.7 ± 43.6	178.1 ± 44.8	181.0 ± 49.4	0.507
Carbohydrate (% kcal)	49.6 ± 9.0	50.5 ± 6.8	53.2 ± 9.4	0.265
Dietary fiber (g)	16.2 ± 6.4	14.3 ± 6.0	16.8 ± 6.1	0.499

^1^ All of the variables are presented as mean ± standard deviation. * Intergroup differences are tested using ANOVA with a post hoc Scheffe test (*p* < 0.05). ^a,b^ Different superscripts indicate statistical difference among the groups. AD—adiposity only; OA–osteopenic/osteoporotic adiposity; OSA—osteosarcopenic adiposity.

**Table 6:** Average daily micronutrient intake including food and supplements in participants according to body composition categories ^1^.

Parameters	AD (*n* = 22)	OA (*n* = 11)	OSA (*n* = 45)	*p* Value *
Vitamins				
Vitamin A (µg RE) ^2^	331.5 ± 264.9	429.0 ± 173.6	458.1 ± 285.2	0.195
Vitamin B_1_ (mg/MJ) ^3^	0.3 ± 0.8	0.5 ± 1.0	0.6 ± 1.5	0.722
Vitamin B_2_ (mg)	1.9 ± 5.4	3.3 ± 7.6	3.6 ± 9.6	0.739
Vitamin B_3_ (mg NE/MJ) ^4^	2.3 ± 1.8	2.4 ± 1.6	2.5 ± 2.1	0.926
Vitamin B_6_ (mg)	2.2 ± 4.3	3.2 ± 6.0	3.7 ± 9.1	0.753
Vitamin C (mg)	151.0 ± 188.4	166.3 ± 146.6	155.8 ± 171.5	0.972
Vitamin D (µg)	1.0 ± 0.4	1.4 ± 0.9	1.1 ± 0.6	0.191
Vitamin E (mg)	6.4 ± 2.1	6.6 ± 2.6	5.0 ± 2.1	0.099
Vitamin K (µg)	54.8 ± 45.4	69.5 ± 48.2	59.1 ± 48.8	0.708
**Minerals**				
Calcium (mg)	534.9 ± 305.2	744.9 ± 417.7	569.5 ± 262.1	0.151
Copper (mg)	0.9 ± 0.4	0.9 ± 0.3	1.0 ± 0.5	0.357
Iron (mg)	7.9 ± 2.4	7.6 ± 2.3	7.4 ± 2.3	0.767
Magnesium (mg)	257.1 ± 195.8	272.0 ± 182.3	245.0 ± 204.9	0.914
Phosphorus (mg)	832.4 ± 236.1	879.8 ± 256.0	759.4 ± 235.1	0.237
Potassium (mg)	1947.0 ± 597.7	2052.0 ± 464.2	1939.9 ± 596.6	0.844
Selenium (µg)	84.6 ± 20.9 ^a^	69.9 ± 15.3 ^b^	71.1 ± 23.4 ^b^	0.048
Sodium (mg)	1575.2 ± 522.5	1769.2 ± 687.7	1546.8 ± 695.4	0.596
Zinc (mg)	5.6 ± 2.7	5.8 ± 2.3	5.9 ± 2.9	0.925

^1^ All of the variables are presented as mean ± standard deviation. ^2^ RE—retinol equivalent (1 µg RE = 1 µg of retinol). ^3^ The thiamin requirement is related to the energy requirement, and is therefore expressed in mg/MJ. ^4^ NE—niacin equivalent (1 NE = 1 mg niacin; niacin requirement is related to energy requirement and is therefore expressed in mg NE/MJ); MJ—megajoule. * Intergroup differences are tested using ANOVA with a post hoc Scheffe test (*p* < 0.05). ^a,b^ Different superscripts indicate statistical difference among the groups. AD—adiposity only; OA—osteopenic/osteoporotic adiposity; OSA—osteosarcopenic adiposity.

## Data Availability

The data are available upon request from I.K. and S.C.
